# Community Health Nurses’ Knowledge and Perceptions of AI in Canada: National Cross-Sectional Survey

**DOI:** 10.2196/78560

**Published:** 2026-01-23

**Authors:** Mary Henderson Betkus, Davina Banner, Leanne Currie, Piper Jackson, Shannon Freeman

**Affiliations:** 1 School of Nursing, University of Northern British Columbia, 3333 University Way, Prince George, BC, V2N 4Z9, Canada, 1 250-613-7507; 2 School of Nursing, University of British Columbia, Vancouver, BC, Canada; 3 Department of Computing Science, Thompson Rivers University, Kamloops, BC, Canada

**Keywords:** registered nurses, community health nurses, artificial intelligence, survey, machine learning

## Abstract

**Background:**

Artificial intelligence (AI) continues to expand into nursing and health care. Many examples of AI applications driven by machine or deep learning are in use. Examples include wearable devices or alerts for risk prediction. AI tends to be promoted by nonnurses, creating a risk that AI is not designed to best serve registered nurses. Community health nurses (CHNs) are a small but essential group. CHNs’ familiarity with AI and their perceptions about its effect on their practice are unknown.

**Objective:**

The research aims to understand CHNs’ awareness, knowledge, and perceptions of AI in practice and gain insights to better involve them in AI.

**Methods:**

An online cross-sectional Canadian survey targeting CHNs was conducted from April to July 2023. Descriptive statistics summarized respondents’ characteristics and perceptions of AI, followed by a chi-square test used to determine a relationship between respondents’ level of AI knowledge and their AI perceptions, with odds ratio (OR) to determine the strength of association.

**Results:**

A total of 228 CHNs participated with varying response rates per question. Most respondents were female (172/188, 91.5%), average age of 45.5 (SD 11.7) years, and an average of 13.5 (SD 10.1) years of community practice experience. Most respondents (205/228, 89.9%) felt they welcomed technology into their practice. They reported their understanding of AI technologies as “good” (95/220, 43.2%) and “not good” (125/220, 56.8%). Overall, 39.6% (80/202) of respondents felt uncomfortable with the development of AI. They agreed that AI should be part of education (143/203, 70.4%), professional development (152/202, 75.2%), and that they should be consulted (195/203, 96.1%). Many respondents had concerns related to professional accountability if they accepted a wrong AI recommendation (157/202, 77.7%) or if they dismissed a correct AI recommendation (149/202, 73.8%). Respondents with “good” AI knowledge were significantly associated with, and twice as likely to indicate nursing will be revolutionized (*P*=.007; OR 2.28, 95% CI 1.25-4.18), nursing will be more exciting (*P*=.001; OR 2.52, 95% CI 1.42-4.47), health care will be more exciting (*P*=.004; OR 2.3, 95% CI 1.30-4.06), and agreed that AI is part of nursing (*P*=.01; OR 2.1, 95% CI 1.19-3.68). Respondents with “not good” AI knowledge were significantly associated with, and more likely to feel uncomfortable with AI developments (*χ*^2^_1_=4.2, *P*=.04; OR 1.84, 95% CI 1.03-3.3).

**Conclusions:**

CHNs reporting “good” AI knowledge had more favorable perceptions toward AI. Overall, CHNs had professional concerns about accepting or dismissing AI recommendations. Potential solutions include educational resources to ensure that CHNs have a sound basis for AI in their practice, which would promote their comfort with AI. Further research should explore how CHNs could be better involved in all aspects of AI introduced into their practice.

## Introduction

### Background

Artificial intelligence (AI) covers a broad array of AI-driven applications supported by machine learning (ML) or deep learning, which have potential utility in health care and nursing practice. Many examples of AI-driven care applications exist, from wearable devices for automated detection of signs and symptoms [1], automated assessment of outcomes to support the need for a different level of care [2,3], client-specific automated predictions of risks [4-7], and bots to answer inquiries and send reminders [8]. Despite widespread uptake and use, AI is commonly driven by nonnurses (ie, scientists, engineers, and the technology industry) [9,10] and physicians [11]. The lack of participation by registered nurses (RNs) creates a risk that AI will not be designed to best serve RNs who are expected to use AI applications and their outcomes in clinical practice [11-13]. Likewise, it is unknown whether community health nurses (CHNs) have thought about how AI applications could change their practice or how AI might be useful to inform clinical practice.

The community setting has a smaller group of RNs compared to the acute care sector [14]. CHNs are RNs who provide essential services in a variety of roles (eg, home health, public health, and primary care) within community settings (eg, clients’ home and schools) [15]. Home health clients are most often older adults with multiple comorbidities [2], or individuals who have chronic [16,17] and unstable conditions [18]. Public health clients can be any age, as the focus of care is on promoting better health with service delivery to groups or individuals [15]. Within these settings, CHNs make the best care decisions based on the information that exists, as well as considering other subtleties that can affect these decisions. Regardless of the setting in the community, CHNs have increased autonomy [15,17,19], and clients have reduced nursing oversight because of time between visits [20]. This decreases the amount and frequency of the client-specific data collected. Further, CHNs focus on human connections and building trustful relationships while recognizing the strengths of individuals and communities to promote and improve their health [15]. These features support the importance of having CHNs who understand the practice area involved in AI.

Nursing research within the community sector is expanding to include a focus on the use of AI (eg, ML) as a method to improve real-time risk predictions [5,20,21] and to assist with better planning or targeting of service delivery [16]. Although involving CHNs would be key to raising the right questions for AI, as well as advising and validating results [22], few researchers are reporting this type of CHN involvement in AI development. More commonly, researchers are using existing collected data [16,21,23]. This passive involvement misses the opportunity of actively involving CHNs who are familiar with the data they collect and how it may add insight to clinical issues. However, in one example, a nonnurse researcher [24] describes using CHNs to advise and evaluate throughout an AI project, concluding that nursing input validated outcomes and facilitated acceptance of the AI algorithm into practice. Hence, nursing involvement provides a relevant perspective and knowledge that influences their informed decisions, which ensures clinical relevance and accuracy of AI and related ML [24,25]. These revelations add impetus to examine CHNs’ perceptions of AI in their practice and to consider how they could be better involved.

### Purpose Statement

This study aims to establish a baseline understanding of Canadian CHNs’ awareness, knowledge, and perceptions of current and future effects of AI on their clinical practice. This will help to gain insights into how CHNs could be better involved in AI. Therefore, the research questions guiding this study include: (1) Are CHNs aware of the emergence of AI, including ML applications, in nursing? (2) What are CHNs’ main sources of knowledge for learning about current day-to-day AI? (3) How do CHNs describe their level of knowledge of AI technologies? (4) Is there a relationship between CHNs’ level of knowledge of AI technologies and their perceptions of the effects of AI on clinical practice, professional accountability, and the usefulness of AI applications? (5) What AI competencies do CHNs perceive as being needed in their community practice?

## Methods

### Ethical Considerations

Research approval was granted from the University of Northern British Columbia (UNBC) Research Ethics Board (REB 6009080), April 2023, to conduct a single cross-sectional open survey using SurveyMonkey licensed through UNBC. The survey landing page included an informational letter to provide study details. After reading the information on the landing page, respondents were asked to voluntarily consent electronically to participate in the study. Upon confirmation of informed consent, participants were then given access to this survey. If a participant did not consent, they received a thank-you message, and access to the survey closed automatically. All aspects of data collection, storage, and analysis were password-protected and housed on an encrypted UNBC server. The invitations advertised a random draw of 5 e-gift cards at the end of the survey period as an incentive to participate in the survey.

### Instrument Design

A total of 11 research papers, which used surveys to examine attitudes and perceptions toward AI, were screened for relevance to this research study’s instrument design. Two papers [26,27] were validating their General Attitudes Artificial Intelligence Scale to classify individuals with positive or negative feelings toward AI. The remaining 9 research studies targeted RNs [28], nursing students [29], radiologists [30,31], physicians [32], medical students [33], a mix of health care professionals [34,35], and consumers [36]. All except Swan [28] had their survey questions included in the publication or supplemental information. A request to preview and use Swan’s survey, if applicable, was granted (BA Swan, RN, PhD, personal communication, November 30, 2022).

Swan’s survey was selected due to its purposeful design for use with nursing professionals. It included similar questions to the other previewed surveys, indicating that common survey topics were covered. Further, Swan’s survey was adapted by adding questions important to this study. In the adapted survey, the first question to address computer expertise was sourced from Schepman and Rodway [27], who suggested that individuals with computer expertise would be more positive about AI. Questions 32 and 33 were added from Esmaeilzadeh [36] with slight modifications to address professional accountability. More details on the survey and its adaptation are found in Multimedia Appendix 1. Swan’s survey had not been tested or piloted before deployment of the survey (BA Swan, RN, PhD, personal communication December 21, 2022).

The revised survey was reviewed for clarity by a retired community nursing manager with over 35 years of community experience, as a public health nurse in direct care and management. It was confirmed that the survey took 20 minutes to complete, and a direct question exploring how nurses should be involved with AI was suggested. Therefore, Q37 “How should nurses be involved in artificial intelligence that influences their practice?” was added.

The final version consisted of 37 content questions (referencing aspects of AI) plus a demographic section, which was used to describe respondents’ representation across Canada, as well as their level of experience and current position. The survey was recreated on the survey platform. Complete wording of each survey item and types of questions are found in Multimedia Appendix 1.

### Recruitment

The target population was RNs licensed in Canada who practiced in the community setting (eg, home care and public health) or RNs who had a community nursing focus (eg, researchers, educators, administrators, and clinical informatic nurses). Collectively, the term CHNs will be used. The survey was only offered in English. The size of the targeted population was unknown. Canadian workforce data reported that 32,074 direct care RNs were employed in community health in 2023 [14]; however, this does not account for the others not providing direct care (eg, researchers, educators, and administrators) included in the population of interest. Therefore, an online calculator [37] was used with the parameters of 20,000 for an unknown population, distribution at 50%, with 5% margin of error and a 95% CI, indicating a sample size of 377 was needed. The emergence of AI into clinical practice remains a new field. Therefore, the power analysis was a reference point to guide this exploratory research study.

The participants were recruited by an “invitation to participate letter,” which had the live link to the survey embedded into its content. This was shared through nursing sources by monthly newsletters, email lists (eg, existing organizational and collegial connections), and informal networks (eg, colleague-to-colleague and social media). Two national organizations, Community Health Nurses of Canada and Canadian Nursing Informatics Association, canvassed their membership by broadcast messages and posts in their monthly e-newsletter. Each provincial and territorial nursing association or licensing body was contacted by email, briefly explaining the research and asking if they would circulate it to their members. Licensing bodies recommended that the researcher contact the nursing associations. One provincial licensing body agreed to send out the invitations by email to their members who identified as working in the community and had previously consented to be contacted for research purposes. The nursing associations kept the invitation in their monthly newsletters, or posts on their social media sites, or sent by broadcast message to their members until the survey closed. The survey was live from April 24 to July 30, 2023.

### Data Management

On the survey closure date, the full dataset was exported from the survey platform to SPSS Statistics (version 29; IBM Corp). All computer IP addresses were removed, as well as respondents who provided consent but did not complete any survey questions. As it was expected that CHNs may complete this survey using a shared workstation, multiple responses from the same IP address were included as long as they were completed at different times, for different durations, and represented unique participant responses. The use of the same IP addresses was limited to 10 instances and met the above criteria. The geographical locations were grouped into regions to determine Canada-wide representation: Eastern (Prince Edward Island, Newfoundland & Labrador, New Brunswick, and Nova Scotia), Central (Ontario and Quebec), Western (Manitoba, Saskatchewan, Alberta, and British Columbia), and Northern (Yukon, Northwest Territories, and Nunavut). Questions that offered “other” as a choice were reviewed and recoded into the appropriate existing choices already provided; otherwise, it was left as “other.” All word responses were coded for a numerical value to enable analysis (eg, Likert scale responses). Surveys that were blank (n=5) were removed. Cases with missing data (greatest in the demographic section) were kept, thus maximizing the number of responses for any given question. Therefore, the count n/N and percent are presented per question, except for multiple response questions, where n values and percent are given, because participants could respond to more than one option. Chi-square analysis was conducted to examine the relationship between respondents’ reported AI knowledge (Q6) and respondents’ perceptions of AI in their practice (Q10-Q20 and Q22-Q35, Q21 “other” was not included). All questions were examined for their missing or incomplete data. Variation in response rates could be due to respondents’ choices not to answer or complete the survey. Therefore, to minimize the potential for response bias, all questions with a less than 15% missing data rate were kept. The core set of survey questions used to examine the research questions met this proportion of missing variables, with response rates as follows: Q6 (220/228, 96.5%), Q10-Q20 (206/228, 90.4% to 208/228, 91.2%), and Q22-Q35 (202/228, 88.6% to 207/228, 90.8%). The missing data for these questions is as follows: Q6 (3.5%), Q10-Q20 (range 8.8% to 9.6%), and for Q22-Q35 (range 9.2% to 11.4%).

### Data Analysis

Both descriptive and inferential statistics were used to examine the data. Descriptive analysis summarized respondents’ characteristics and their perceptions of AI in nursing. Inferential statistics examined the relationship between their reported AI knowledge (Q6) and their perceptions of the current and future effects of AI on nursing and health care.

The chi-square test for independence was used to determine an association between CHNs reported level of knowledge of AI technologies (independent variables) and their perceptions of the effects of AI (dependent variables). The CHNs were grouped by their reported level of knowledge of AI technologies to allow for comparison. CHNs described their level of knowledge of AI technologies as “excellent,” “very good,” “good,” “fair,” or “none.” They were grouped as “good” level of knowledge if they indicated “good” to “excellent” and “not good” level of knowledge if they indicated “fair” or “none.” The reference category chi-square test for independence was a primarily “good” level of AI knowledge; however, a “not good” level of AI knowledge was the reference category for Q26 (comfort with AI development), Q32 (concern with AI offering wrong recommendation), and Q33 (concern with dismissing appropriate AI recommendation) to promote ease in explaining the results. All statements related to CHNs’ perception or attitudes about AI were a 5-point Likert scale from strongly agree (5), agree (4), neutral (3), disagree (2), and strongly disagree (1). The responses for these questions were grouped as “agree” if the respondent indicated “agree” or “strongly agree” and grouped as “not agree” if they indicated “neutral,” “disagree,” or “strongly disagree.” Neutral was grouped with “not agree” because it was interpreted that this group of respondents had no definitive feeling either way on the subject. As the aim of the research was to gain an understanding of how to better involve nurses in AI, it was concluded that these “neutral” respondents, along with “not agree,” may need more targeted strategies to better involve them. Further, the transformed response “agree or not agree” was clarified by the sentiment being examined to ease understanding. The dependent variables were considered: comfortable or not comfortable with AI development, AI applications useful or not useful, effects of AI agree or not agree, and professional accountability concerned or not concerned. Odds ratios were calculated for chi-square tests that were significant to determine the strength of association.

Correction (ie, Yates and Bonferroni) methods for statistical testing were not used. Yates continuity correction was not used because the sample size was considered large enough (range 202 to 208) to support a Pearson chi-square [38]. It is noted that the item “nurse should be consulted” produced cells under 5 (not agree); however, this seemed a reasonable result and would not benefit from Yates correction. The Bonferroni post hoc was not used because it can be too restrictive [39]. The Bonferroni post hoc (0.05/25=0.002) is given for reference only and includes the 25 items (Q10-Q20 and Q22-Q35) examined for association.

The open-text question asking the respondents “How should registered nurses be involved in AI?” was examined for types of responses. Some examples of these responses included how CHNs could be engaged in AI technologies, for example, education, advising, or consulting. These responses were quantified with the frequencies reported.

## Results

### Overview

A total of 296 potential respondents opened the survey, 261 met recruitment criteria, with 233 (89.3%) providing consent. As reported, blank surveys (n=5) were not included. A total of 228 surveys were included in the analyses. The response rate fluctuated per question, with the response rate better at the start of the survey and waning by the final demographic section. The item, “community years experience,” had the most nonresponses (52/228, 22.8%).

### Sample Characteristics

Sample characteristics (Table 1) helped to describe the sample that responded to the survey. The respondents’ average age was 45.5 (SD 11.7) years, with 58.4% (104/178) younger than 50 years. Most respondents identified as female (172/188, 91.5%). The average overall years of experience for RNs was 19.8 (SD 12.2) years, with most (161/179, 89.9%) ranging from 5 years to over 35 years of experience. For community practice, the average years of experience was 13.5 (SD 10.1) years, with many (129/176, 73.3%) ranging from 5 years to over 35 years of experience. The sample had representation from the 4 Canadian regions: Eastern (47/186, 25.3%), Central (72/186, 38.7%), Western (65/186, 34.9%), and Northern (2/186, 1.1%). The practice descriptions are multiple-response questions. The reported practice settings (Table 2) included public health (65/191, 22.3%), home care (56/191, 19.2%), community health centers (44/191, 15.1%), primary care (41/191, 14%), and case management (16/191, 5.5%). Approximately half indicated they provided direct care (108/191, 51.4%), and the majority (115/190, 60.5%) held a bachelor’s degree.

**Table 1.** Sample characteristics of respondents.

Characteristic	Participants
Gender, n (%)^a^	
Male	16 (8.5)
Female	172 (91.5)
Age (years), means (SD)	45.5 (11.7)
Age (years), n (%)^b^	
25-29	13 (7.3)
30-34	26 (14.6)
35-39	29 (16.3)
40-44	17 (9.6)
45-49	19 (10.7)
50-54	30 (16.9)
55-59	21 (11.8)
60 and older	23 (12.9)
RN^c^ experience (years), means (SD)	19.8 (12.2)
RN experience (years), n (%)^d^	
Less than 5 years	18 (10.1)
5-9	24 (13.4)
10-14	29 (16.2)
15-19	22 (12.3)
20-24	18 (10.1)
25-29	21 (11.7)
30-34	22 (12.3)
35 and greater	25 (14)
Community experience (years), means (SD)	13.5 (10.1)
Community experience (years), n (%)^e^	
Less than 5 years	47 (26.7)
5-9	24 (13.6)
10-14	30 (17)
15-19	26 (14.8)
20-24	13 (7.4)
25-29	19 (10.8)
30-34	13 (7.4)
35 and greater	4 (2.3)
Geographic location, n (%)^f^	
Eastern Canada	47 (25.3)
Central Canada	72 (38.7)
Western Canada	65 (34.9)
Northern Canada	2 (1.1)

a N=188.

b N=178.

c RN: registered nurse.

d N=179.

e N=176.

f N=186.

**Table 2.** Education and employment data of respondents.

Characteristic	Participants
Education level, n (%)^a^	
Diploma	27 (14.2)
Bachelor	115 (60.5)
Masters	38 (20)
Doctoral or PhD	10 (5.3)
Employment sector, n (%)^b^,^c^	
Public	140 (70.4)
Private	45 (22.6)
Academia	14 (7)
Practice setting, n (%)^b^,^d^	
Health informatics	12 (4.1)
Community health	44 (15.1)
Case management	16 (5.5)
Older adult	13 (4.5)
Home care	56 (19.2)
Hospice palliative	11 (3.8)
Primary care	41 (14)
Community mental health	9 (3.1)
Public health	65 (22.3)
College or university	18 (6.2)
Other	7 (2.4)
Current position (years), n (%)^b^,^d^	
Direct care	108 (51.4)
Nurse informatician	6 (2.9)
Manager or administrator	34 (16.2)
Staff education	23 (11)
Researcher	6 (2.9)
Faculty	19 (9)
Strategic planning	5 (4.3)
Other	9 (4.3)

a N=190.

b Multiple response questions, n summed in each section, may be greater than N.

c N=187.

d N=191.

### Acceptance of Technology and Competent Users of Technology

The survey questions 1 and 2 were used to explore the CHNs’ acceptance of technology into their practice, and how they described their computer use. Almost all participants (205/228, 89.9%) agreed or strongly agreed to welcoming technology into their practice. More than half (129/227, 56.8%) identified as competent users of the internet and standard applications, and another 36.6% (83/227) indicated they were users of specialist applications. The survey, included in Multimedia Appendix 1, is subdivided into sections related to the headings addressing each of the research questions.

### CHNs’ Awareness of the Emergence of AI, Including ML Applications, in Nursing

CHNs’ awareness of AI (Q8) in health care was more prevalent than their awareness of AI in nursing. The respondents were aware of AI (multiple response questions) in health care (123/220, 55.9%), but fewer were aware of AI in nursing (67/220, 30.5%). This was similar for ML and deep learning (Q9): respondents had heard of it in health care (84/220, 38.2%), and fewer had heard of it in nursing (35/220, 15.9%).

### CHNs’ Main Sources of Knowledge for Learning About Current Day-to-Day AI

**Figure 1.**
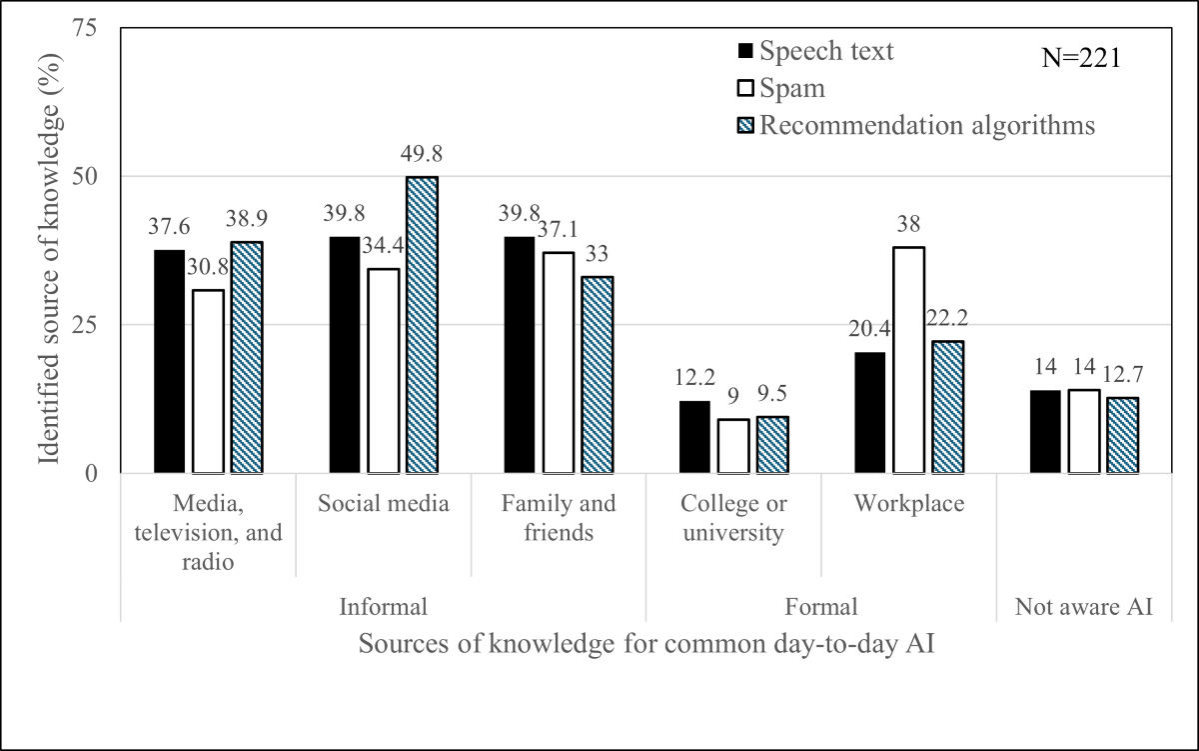
Respondents’ key sources of knowledge for day-to-day artificial intelligence. AI: artificial intelligence.

The key sources of knowledge for learning about current day-to-day AI (Q3-Q5) varied between informal and formal methods. The respondents’ major source of knowledge (multiple response questions) of common forms of day-to-day AI applications (Figure 1; ie, speech-text, spam, and recommendation algorithms) was informal resources such as media, television, or radio (range 68/221, 30.8% to 86/221, 38.9%); social media (range 76/221, 34.4% to 110/221, 49.8%); and family and friends (range 73/221, 33% to 88/221, 39.8%). Formal sources were indicated less often: colleges and universities (range 20/221, 9% to 27/221, 12.2%) and workplace (range 45/221, 20.4% to 84/221, 38%). It is worth noting that some respondents were not aware that these applications (speech-to-text, spam, and recommendation algorithms) were forms of AI (range 28/221, 12.7% to 31/221, 14%).

### CHNs’ Description of Their Level of Knowledge of AI Technologies

Respondents described their understanding of the technologies used in AI (Q6) as none (42/220, 19.1%), fair (83/220, 37.7%), good (67/220, 30.5%), very good (23/220, 10.5%), and excellent (5/220, 2.3%). These results were grouped into 2 levels of AI knowledge: “good” level of knowledge included good to excellent (95/220, 43.2%), and “not good” level of knowledge used fair and none (125/220, 56.8%). Level of AI knowledge (Q6) was used in the chi-square test, as AI was more commonly known with a more balanced representation. For ML or deep learning (Q7), it was a similar trend, more nurses indicated “not good” level of knowledge (148/220, 67.3%) than “good” level of knowledge (72/220, 32.7%).

### The Relationship Between CHNs’ Level of Knowledge of AI Technologies and Their Perceptions of the Effects of AI on Clinical Practice, Professional Accountability, and the Usefulness of AI Applications.

#### Effects of AI on Clinical Practice

Questions 22 to 31 and 34 to 35 examined the respondents’ perception of the effects of AI on their practice. An overview of respondents’ perceptions indicated 39.6% (80/202) felt uncomfortable with the developments in AI, ML, and deep learning. Over half (133/206, 64.6%) of the respondents agreed that AI would revolutionize both health care and nursing. Few respondents agreed that the human nurse, 10.2% (21/205), or members of the interprofessional team, 12.6% (26/207), would be replaced. Almost half of respondents felt AI would make nursing more exciting, 44.1% (89/202), and similarly, health care more exciting, 47.5% (96/202). Likewise, 44.8% (91/203) perceived AI to be part of nursing. Many respondents (143/203, 70.4%) felt that AI should be part of nursing education and included in professional development (152/202, 75.2%). Most respondents agreed they should be consulted (195/203, 96.1%) about AI, as well as having the opportunity to raise relevant nursing questions (189/202, 93.6%).

Examination with the chi-square test for independence (Table 3) was used to determine if there was a relationship between the respondents’ reported AI knowledge and their perceptions of the potential effects of AI on clinical practice. For Q26, the reference category for level of AI knowledge was “not good.” There was a significant relationship between respondents reporting “not good” level of AI knowledge and their perception of “feel uncomfortable” (ie, “agree” with statement) with AI developments (*χ*^2^_1_=4.2, *P*=.04; *α*=.05; small effect *φ*=.15). Respondents reporting “not good” AI knowledge were 1.84 (95% CI 1.03-3.3) times more likely to indicate developments in AI made them feel uncomfortable.

**Table 3.** Respondents’ perceptions of current and future effects of AI^a^ on clinical practice related to their level of AI knowledge.

Questions	Knowledge level	Effects		Chi-square (*df*)	Effect (φ)	OR^b^ (95% CI)	*P* value
		Agree n (%)	Not agree n (%)				
Q22 revolutionize nursing				7.3 (1)	0.19	2.28 (1.25-4.18)	.007
	Good	66 (75)	22 (25)				
	Not good	67 (56.8)	51 (43.2)				
Q23 revolutionize health care				2.3 (1)	0.11	1.58 (0.88-2.86)	.13
	Good	62 (70.5)	26 (29.5)				
	Not good	71 (60.2)	47 (39.8)				
Q24 replace human RN^c^				0.85 (1)	0.07	1.53 (0.62-3.78)	.36
	Good	11 (12.5)	77 (87.5)				
	Not good	10 (8.5)	107 (91.5)				
Q25 replace interprofessional team member				0.2 (1)	0.03	1.18 (0.52-2.70)	.69
	Good	12 (13.6)	76 (86.4)				
	Not good	14 (11.8)	105 (88.2)				
Q26 uncomfortable with AI developments^d^				4.2 (1)	0.15	1.84 (1.03-3.3)	.04
	Not good	53 (45.7)	63 (54.3)				
	Good	27 (31.4)	59 (68.6)				
Q27 nursing will be more exciting				10.1 (1)	0.22	2.52 (1.42-4.47)	.001
	Good	49 (57)	37 (43)				
	Not good	40 (34.5)	76 (65.5)				
Q28 health care will be more exciting				8.3 (1)	0.20	2.3 (1.30-4.06)	.004
	Good	51 (59.3)	35 (40.7)				
	Not good	45 (38.8)	71 (61.2)				
Q29 AI is part of nursing practice				6.6 (1)	0.18	2.1 (1.19-3.68)	.01
	Good	48 (55.2)	39 (44.8)				
	Not good	43 (37.1)	73 (62.9)				
Q30 AI included in nursing education				0.7 (1)	0.06	1.3 (0.71-2.4)	.40
	Good	64 (73.6)	23 (26.4)				
	Not good	79 (68.1)	37 (37.9)				
Q31 AI included in professional development				2.0 (1)	0.10	1.6 (0.83-3.14)	.16
	Good	69 (80.2)	17 (19.8)				
	Not good	83 (71.6)	33 (28.4)				
Q34 nurses should be consulted				0.2 (1)	−0.03	0.74 (0.18-3.05)	.68
	Good	83 (95.4)	4 (4.6)				
	Not good	112 (96.6)	4 (3.4)				
Q35 identify relevant AI nursing questions				2.0 (1)	−0.10	0.44 (0.14-1.39)	.15
	Good	78 (90.7)	8 (9.3)				
	Not good	111 (95.7)	5 (4.3)				

a AI: artificial intelligence.

b OR: odds ratio.

c RN: registered nurse.

d Reference category was set to ”good” for all variables with the exception of Q26 where the reference category was set to “not good.”

The remaining statements (Q22-Q25 and Q27-Q35) used the reference category “good” level of AI knowledge. There were significant relationships between “good” level of AI knowledge and the following perceptions. Respondents perceived AI would revolutionize nursing (*χ^2^*_1_=7.3, *P*=.007; *α*=.05; small to moderate effect *φ*=.19) and were 2.28 times more likely to agree that nursing would be revolutionized (95% CI 1.25-4.18). Respondents perceived AI would make both nursing (*χ^2^*_1_=10.1, *P*=.001, *α*=.05, small to moderate effect *φ*=.22) and health care (*χ^2^*_1_=8.3, *P*=.004, *α*=.05, small to moderate effect *φ*=.20) more exciting. Respectively, these respondents were 2.52 times more likely (95% CI 1.42-4.47) and 2.3 times more likely (95% CI 1.30-4.06) to perceive that AI will make nursing and health care more exciting. These respondents perceived that AI is part of nursing practice (*χ^2^*_1_=6.6, *P*=.01; *α*=.05; small to moderate effect *φ*=.18) and were 2.1 times more likely to agree that AI is part of nursing practice (95% CI 1.19-3.68).

There was no association observed between level of AI knowledge and perceived effects: for revolutionizing health care (*P*=.13) nor between level of AI knowledge and perceived effects for replacing human RN (*P*=.36) or replacing interprofessional team members (*P*=.69). There was no association between level of AI knowledge and perception that AI should be part of nursing education (*P*=.4), part of professional development (*P*=.16), nurses should be consulted (*P*=.68), or nurses should identify relevant nursing questions for AI (*P*=.15).

#### Professional Accountability

Two statements (Q32 and Q33) used “what if” scenarios to examine CHNs’ perceptions of AI and professional accountability. One described an AI providing the wrong recommendation, and the other described a correct recommendation that was dismissed by the nurse. Respondents expressed concern regarding their responsibility in both scenarios. The majority, 77.7% (157/202), were concerned if AI offered the wrong recommendation, and likewise, 73.8% (149/202), if an appropriate AI recommendation was dismissed. Examination with chi-square test for independence (Table 4) with “not good” as the reference category revealed no association between level of AI knowledge and perceived concern if AI provided a wrong recommendation (*P*=.06). Conversely, the chi-square test for independence suggested a significant association between a “not good” level of AI knowledge and perceived concern if a correct recommendation was dismissed (*χ*^2^_1_=3.98, *P*=.046; *α*=.05; small effect *φ*=.14). Respondents reporting “not good” AI knowledge were 1.9 times more likely to be concerned with dismissing an appropriate AI recommendation (95% CI 1.01-3.57).

**Table 4.** Respondents’ perceptions of concern with professional accountability related to their level of AI^a^ knowledge.

Questions	Knowledge level	Concern		Chi-square (*df*)	φ	OR^b^ (95% CI)	*P* value
		Agree n (%)	Not agree n (%)				
Q32 if AI offers wrong recommendations				3.7 (1)	0.14	1.9 (0.98-3.74)	.06
	Not good	95 (82.6)	20 (17.4)				
	Good	62 (71.3)	25 (28.7)				
Q33 if correct recommendation is dismissed				3.98 (1)	0.14	1.9 (1.01-3.57)	.046
	Not good	91 (79.1)	24 (20.9)				
	Good	58 (66.7)	29 (33.3)				

a AI: artificial intelligence.

b OR: odds ratio.

#### Usefulness of AI Applications

**Figure 2.**
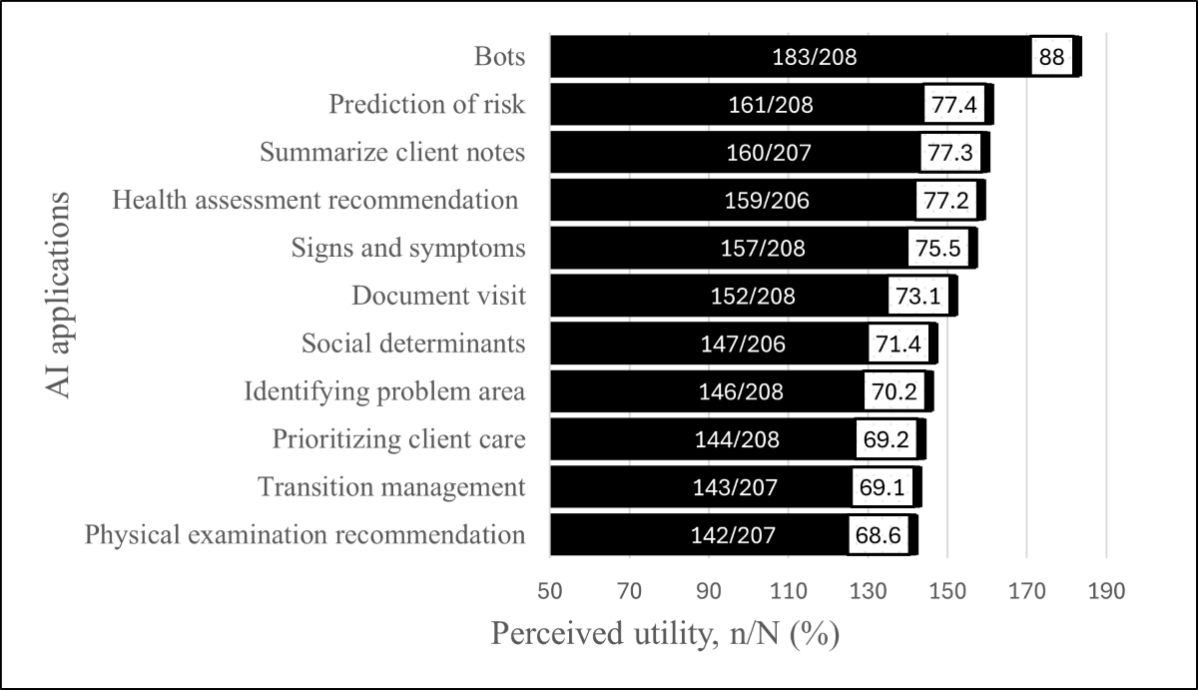
Respondents’ perception of the utility of AI applications. AI: artificial intelligence.

Q10-Q20 examined the respondents’ perceptions of the utility of various AI applications. Respondents perceived that overall, each AI application would be useful (Figure 2), with agreement ranging from 68.6% (142/207) to 88% (183/208). Most respondents indicated Q15 bots (183/208, 88%), Q18 risk prediction (161/208, 77.4%), and Q20 summarizing narrative text from a client’s notes (160/207, 77.3%) would be useful. Further examination to determine if the level of AI knowledge was associated with CHNs’ perception of the utility of AI application revealed that in all but one example, there was no association between level of AI knowledge and their perception of utility (Table 5). There was a significant association between a “good” level of AI knowledge and perception of utility for Q13 transition management (*χ^2^*_1_=7.9, *P*=.005, *α*=.05, small to moderate effect *φ*=.2). Respondents reporting “good” AI knowledge were 2.45 times more likely to agree that transition management would be useful (95% CI 1.3-4.63).

**Table 5.** Respondents’ perception of the utility of AI^a^ applications related to their level of AI knowledge.

Questions	Knowledge level	Utility of AI		Chi-square (*df*)	φ	OR^b^ (95% CI)	*P* value
		Agree n (%)	Not agree n (%)				
Q10 signs and symptoms				0.27 (1)	0.034	1.18 (0.62-2.26)	.61
	Good	68 (77.3)	20 (22.7)				
	Not good	89 (74.2)	31 (25.8)				
Q11 social determinants				0.8 (1)	0.06	1.3 (0.72-2.48)	.36
	Good	65 (74.7)	22 (25.3)				
	Not good	82 (68.9)	37 (31.1)				
Q12 prioritizing client care				0.9 (1)	0.07	1.3 (0.73-2.45)	.35
	Good	64 (72.7)	24 (27.3)				
	Not good	80 (66.7)	40 (33.3)				
Q13 transition management				7.9 (1)	0.20	2.45 (1.30-4.63)	.005
	Good	70 (79.5)	18 (20.5)				
	Not good	73 (61.3)	46 (38.7)				
Q14 problem area				3.7 (1)	0.13	1.83 (0.98-3.42)	.06
	Good	68 (77.3)	20 (22.7)				
	Not good	78 (65)	42 (35)				
Q15 bots				0.03 (1)	−0.01	0.93 (0.40-2.15)	.86
	Good	77 (87.5)	11 (12.5)				
	Not good	106 (88.3)	14 (11.7)				
Q16 health assessments				0.1 (1)	−0.02	0.90 (0.47-1.74)	.76
	Good	67 (76.1)	21 (23.9)				
	Not good	92 (78)	26 (22)				
Q17 physical assessment				0.04 (1)	−0.01	0.94 (0.52-1.7)	.84
	Good	59 (67.8)	28 (32.2)				
	Not good	83 (69.2)	37 (30.8)				
Q18 prediction of risk				0.4 (1)	0.04	1.24 (0.64-2.41)	.53
	Good	70 (79.5)	18 (20.5)				
	Not good	91 (75.8)	29 (24.2)				
Q19 documentation of visit				3.2 (1)	0.13	1.8 (0.95-3.44)	.07
	Good	70 (79.5)	18 (20.5)				
	Not good	82 (68.3)	38 (31.7)				
Q20 summarize client notes				1.6 (1)	0.09	1.54 (0.78-3.05)	.21
	Good	71 (81.6)	16 (18.4)				
	Not good	89 (74.2)	31 (25.8)				

a AI: artificial intelligence.

b OR: odds ratio.

### AI Competencies CHNs Perceive as Being Needed in Their Community Practice

**Figure 3.**
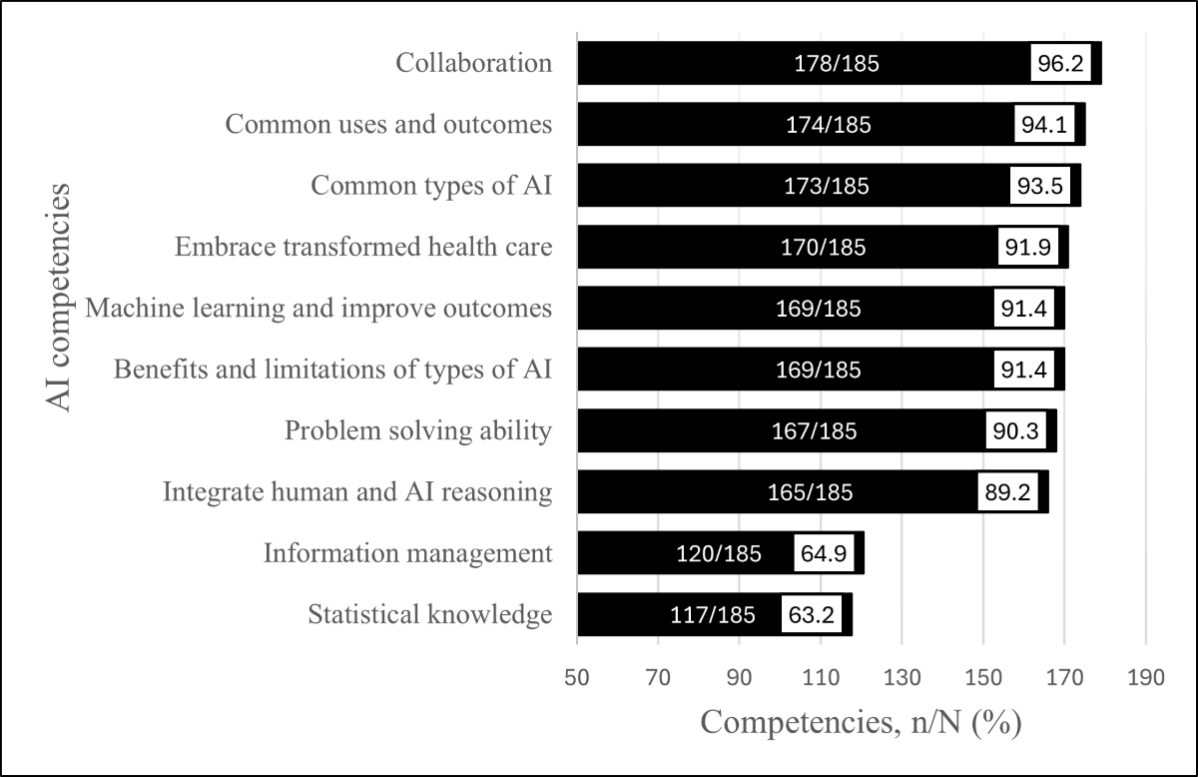
Competencies identified by respondents as needed by CHNs to integrate AI into clinical practice. AI: artificial intelligence; CHN: community health nurse.

The survey (Q36) offered 10 competencies for respondents to indicate which were needed by CHNs (multiple-response question). The 3 competencies most identified as needed (Figure 3) were (1) communications, collaboration, and cross-functional knowledge (178/185, 96.2%); (2) knowledge of common uses and outcomes of AI (174/185, 94.1%); and (3) knowledge of common types of AI (173/185, 93.5%). The competency identified least was statistical knowledge, which also covered skills related to clinical analytics, data management, and algorithm awareness (117/185, 63.2%). Complete wording of each competency is found in Multimedia Appendix 1.

### Insights on How CHNs Could Be Better Involved in AI

An open-ended question (Q37) asked how respondents thought they should be involved. It produced (70/228, 30.7%) responses, which provided insights into nurse involvement and their perspectives on related aspects of AI in their practice. Respondents expressed a need for further education (21/70, 30%) using phrases like “learn,” “knowledge acquisition,” “stay up to date,” and “education.” Most respondents (57/70, 81.4%) cited numerous roles or functions where nurses should be involved: raising relevant questions (5/70, 7.1%); advising and consulting (24/70, 34.3%); planning, development, and implementing (14/70, 20%); evaluation (12/70, 17.1%); change management (5/70, 7.1%); regulation, policy, and ethics (7/70, 10%); and all phases (13/70, 18.6%). They used terms like “key stakeholders” and “subject matter experts.” They felt nurses needed to be involved to make AI relevant. Respondents (7/70, 10%) specifically identified that direct care (front-line and end user) CHNs should be involved. Some respondents (8/70, 11.4%) referred to AI as a tool or an additional resource. Other respondents (13/70, 18.6%) acknowledged their apprehension with AI being introduced into practice. Respondents (6/70, 8.6%) referred to the need to be mindful about the human relationship with phrases like “relationships are key aspects of community health nursing” and “human connection care can not be replaced.”

## Discussion

### Principal Findings

The main findings indicated CHNs differ in their level of knowledge and perceptions of AI technologies in nursing and health care. Many CHNs have a limited awareness of AI emerging in health care and report even less awareness of AI emerging in nursing practice. The main sources of information for day-to-day AI applications are predominantly informal methods (eg, social media) compared to academic and workplace sources. Some CHNs are unaware that common day-to-day applications are AI-driven. Fewer CHNs describe their knowledge of AI technologies as “good.” However, the CHNs who describe their AI knowledge as “good” are twice as likely to be optimistic or have favorable perceptions of AI effects, such as revolutionizing nursing, making nursing more exciting, and agreeing that AI is part of nursing. Whereas CHNs with “not good” AI knowledge are almost twice as likely to feel uncomfortable with AI development. Regardless of the level of AI knowledge, most CHNs agree they should be involved in AI by consulting and raising nurse-relevant questions in various phases of AI development, such as implementation and ongoing evaluation. The results substantiate the need for appropriate AI education for CHNs to prepare them to participate in AI that will influence their practice.

CHNs have a limited awareness of AI emerging in nursing practice (30.5%), which aligns with results found in similar nursing research [28,40]. However, other questions in this current research are used to gain further insights into why their understanding of various AI technologies might be limited. CHNs use informal (eg, social media, and family and friends) methods of learning about common day-to-day AI applications, with 12.7% (28/221) to 14% (31/221) of respondents being unaware that these common forms (ie, speech to text, spam, and recommendation algorithms) are driven by AI. This limited awareness could be related to relying on informal sources of knowledge. CHNs may turn to readily available sources of information because of convenience. Likewise, being aware of spam from work-related sources could be as simple as “don‘t see a reply, check your spam folder,” while having no real understanding of the algorithms that recognize and reroute spam. This lack of understanding whether an application is driven by AI has been linked to clinical practice by another study [40] where 22% of Canadian nurses did not know if AI is used in their practice area. Similarly, Coakley et al [31] identified that approximately 40% of radiographers did not recognize work-related AI-driven applications. This raises a potential concern that CHNs may be using AI-driven applications within their practice unbeknownst to them. Lastly, over half (125/220, 56.8%) of CHNs describe their knowledge of AI technologies as “not good.” This limited awareness of AI in nursing and lack of knowledge of AI technology highlights a knowledge deficit, stressing the importance of AI education for CHNs.

The composition of the survey sample strengthens the clinical value of the results. First, this Canadian sample is an experienced group of CHNs, both in years of practice as an RN and years of experience in the community sector. They describe themselves as competent and welcoming of technology. This was expected because Canada has been striving since 2000 to improve digital health connections (eg, electronic health records) within the Canadian health care system [41]. A current report [40] confirms a continual uptake in digital technology. This steady increase of new technologies into practice (eg, electronic health records and electronic assessments) emphasizes CHNs’ adaptability and resiliency to new technologies in their practice, considering these decisions are made at higher levels in the organization rather than from staff who are expected to use them [42]. Second, more than half of the survey respondents provide direct care services. This means they are familiar with community practice, its clinical data, and provision of care at the client level, and have the potential to offer pragmatic insights. Third, this group of CHNs includes end users who are seldom involved in the development of AI. They are, however, important stakeholders in ensuring clinical relevance in new technology [22,24]. The various characteristics (eg, experienced, competent, and end users) of this CHN sample provide validity and relevance to the results.

A common technique for assessing the level of knowledge across surveys is asking the respondent to indicate their level of AI knowledge. Most surveys use this subjective method, finding fewer respondents rate their level of AI knowledge as “good” compared to “not good” level of knowledge) [28,32,40,43,44], aligning with the current study (level of knowledge “good” 95/220, 43.2% versus “not good” 125/220, 56.8%). None of the cited surveys uses the difference in knowledge level to compare groups and their perceptions.

The subjective evaluation of CHNs’ level of AI knowledge may be underestimated or overestimated. However, professionally, CHNs self-reflect on practice and learning gaps, so they have familiarity in evaluating their competencies [45]. It seems plausible to use the self-identified AI knowledge level as a starting point to determine if there is a relationship between the level of knowledge and CHNs’ perceptions of AI. The 2 groups of “good” and “not good” knowledge level of AI technologies in this study suggest that the level of AI knowledge affects some of the AI perceptions of CHNs.

The CHNs reporting “not good” level of knowledge are almost two times more likely to indicate that they are uncomfortable with the developments in AI. Intuitively, this makes sense. It can be argued that having “good” AI knowledge provides a method to evaluate the benefits or disadvantages of AI and perhaps provides some control [46]. CHNs reporting a “good” level of knowledge are more than two times more likely to feel nursing will be revolutionized, nursing and health care will become more exciting, and agree that AI is part of nursing practice. Therefore, CHNs with a “good” level of AI knowledge are more optimistic about the future effects of AI [46]. The differences between AI perceptions for CHNs with a “good” level of knowledge versus a “not good” level further stress the necessity for education and ongoing learning opportunities to decrease apprehension and promote optimism around AI [46].

Regardless of their level of knowledge, few CHNs believe that human RNs (21/205, 10.2%) or interprofessional team members (26/207, 12.6%) will be replaced by AI. This sentiment aligns with that of Swan [28]. The underlying belief that human touch is integral to nursing care, along with humans’ ability to reconsider and change care when an unexpected situation arises, supports human RNs and other interprofessional members’ continued importance to the care team [47-49]. CHNs’ responses (Q37) defend the importance of human involvement: “relationships are a key aspect of community health nursing” and “human connection care can not be replaced.” CHNs’ belief that they will not be replaced does not address how they think their role within health care will change. This aspect should be examined in future research.

Professional accountability is a central feature for all regulated professionals. Several studies include some reference to the issue (eg, medical liability). This current study demonstrates a mixed outcome. There is no association between the level of AI knowledge and concern with AI providing a wrong recommendation, versus an association between the level of AI knowledge and concern with dismissing a correct recommendation. Still, the clinical importance should be addressed because most CHNs, regardless of their level of AI knowledge, have concerns about who would be held responsible for either accepting a wrong recommendation (157/202, 77.7%) or dismissing a correct recommendation (149/202, 73.8%). Other studies confirm that professionals have concerns about the use of AI in practice [32,40,43]. Further research needs to explore what CHNs feel they require to help address and remediate their concerns.

Although CHNs have a limited understanding of AI, more than two-thirds perceived the examples of AI-driven applications as useful. It suggests that, superficially, they perceived a value in the application to their practice setting. Positive perceptions of the utility of AI applications trend across surveys [28,33,40,43]. However, it is unknown why CHNs perceive the AI applications as useful to them; that is, whether it is the function of replacing a task or the function of supporting decision-making that is important. Future research should follow the open text response “start by asking nurses what they feel could be automated,” thus gaining an understanding of what makes an application useful.

A more comprehensive picture of how CHNs should be involved in AI becomes apparent through the open text responses. First, CHNs confirm their need to “learn,” for “knowledge acquisition,” to “stay up to date,” and “education” as important prerequisites to being involved in AI. Although the quantitative sample identifies the necessity of AI to be included in nursing education and professional development, the open text responses connect AI education to facilitating CHNs’ involvement (participation). Second, the open text responses share a wide range of ways CHNs can be involved. It verifies the importance of including all levels of nurses, specifically noting direct care nurses, and validates that CHNs need to be engaged during all phases of AI. Areas of involvement include: raising relevant questions, planning, development, implementing, evaluation, and monitoring to ensure AI is clinically relevant and accurate. Third, they recognize that their involvement includes regulation, workplace policies, and ethical frameworks to guide their practice because AI is a tool. Fourth, they readily admit to being apprehensive, citing concerns with loss of some of their skills (eg, assessment) along with the human connection and relationship with clients because of AI. This loss of human connection because of technology is also a common concern explored in the literature [49,50]. Further research should continue to examine how CHNs can be better involved.

### Clinical Implications and Recommendations

This research reveals 2 interrelated concepts, preparation and participation. These are both essential to better involve CHNs. The first, preparation, acknowledges the importance of education and ongoing professional development. This builds the foundation that will support CHNs to become involved. AI needs to be consciously integrated both in nursing education and ongoing professional development, with attention to a standardized curriculum to ensure all nurses have a basic understanding of AI. Specific areas of concentration should address professional accountability. This will provide CHNs with knowledge to evaluate AI outputs as part of their decision-making, as well as planning and ameliorating perceived future effects of AI. The second, participation, addresses the various aspects of involving CHNs to identify relevant questions and to contribute their nursing perspective to all phases of development and implementation of AI. Professional nursing groups and health care organizations are instrumental in ensuring that the right mix of CHNs, from end user to leadership, have participation on AI advisory committees. Although this research was initiated to examine the perceptions of CHNs about AI in clinical practice, it now raises the necessity of further research to expand on these results by conducting small group consultations to gain an in-depth understanding of how best to involve CHNs.

### Strengths and Limitations

The strengths of this study include establishing baseline knowledge and perceptions of AI among Canadian CHNs. An effort was made to recruit the appropriate sample population by targeting national nursing groups as well as provincial and territorial nursing organizations. This survey identifies the need for appropriate education (preparation) and confirms that CHNs want to be involved (participation). It explores the use of the self-reported level of knowledge to determine differences between the “good” and “not good” levels of knowledge.

Several limitations exist. The original survey that was foundational to this study did not have psychometric or reliability testing done. Further testing and reporting of reliability is recommended as a future step. In this variation of the survey, only 1 experienced community nurse was used to determine face validity. A notable limitation of the research is the length of the instrument, as nonresponses increased as the survey progressed. The multiple response questions allowed for several responses, which may have blurred the interpretation. For example, under “current position,” a respondent could have 2 different positions within the community, for example, direct care and educator. Self-reported knowledge is subjective; we are unable to verify what the respondents know or do not know or evaluate the expertise of their knowledge. However, CHNs who feel they have knowledge were more favorable or optimistic about AI within their practice. This study did not examine whether respondents had AI-related practical experience or whether current AI is integrated into their practice. Each survey statement or question is briefly explained or described, for example, “AI will revolutionize nursing by supporting health promotion and disease prevention, helping create personalized treatment plans, speeding up administrative tasks.” Each respondent could interpret it differently depending on their understanding of how this might occur and their experience with any of the concepts in the descriptor. There were no respondents identified from Prince Edward Island, Saskatchewan, or the Northwest Territories. With a nonresponse rate (42/228, 18.4%) for this question, it could not be determined where the missing respondents were located. An online survey has challenges. The recommended number of 377 respondents to have a 5% error margin was not achieved. The true response rate is unknown because it is unknown how many CHNs received the recruitment invitations due to the method used to recruit respondents. Respondents’ bias or selective reporting may have occurred because it was an online survey; only nurses who could access the survey could respond. Additionally, using the term AI in the survey title may have only interested a select group of nurses. As well, the survey was only offered in English, limiting the participation and insights from Francophone nursing colleagues. Lastly, for analysis, the chi-square test can only test for the association of the categorical variables, not causation.

### Conclusions

The survey results provide insights into the proposed research questions. Only a third of CHNs are aware that AI is emerging in nursing practice. CHNs use informal sources of knowledge (eg, family and friends) to learn about day-to-day AI applications, with some unaware that these day-to-day applications are AI-driven. This raises the concern that CHNs may be using AI in their practice without realizing that the technology they are using is AI-based. CHNs who report better AI knowledge tend to be more optimistic (ie, “more exciting”) and less uncomfortable about AI and its effects on practice. However, many CHNs have concerns with AI and their professional accountability. Many CHNs agree that AI as a topic should be included in nursing education as well as professional development. This study identifies that most CHNs want to be involved in AI, highlighting that they want to be consulted and given opportunities to raise nurse-relevant questions. An important step to better involve CHNs should address the availability of appropriate and consistent education. This will help to promote the awareness of AI in nursing and alleviate professional concerns, thus preparing CHNs to be better involved.

## Supplementary material

10.2196/78560 Multimedia Appendix 1 Survey instrument.

10.2196/78560 Checklist 1 CHERRIES checklist.

## Abbreviations:

AI: artificial intelligence
CHN: Community Health Nurse
ML: machine learning
OR: odds ratio
RN: registered nurse
UNBC: University of Northern British Columbia
