# Supplementary material for: Community Health Nurses’ Knowledge and Perceptions of AI in Canada: National Cross-Sectional Survey
Source: JMIR Nurs. 2026 Jan 23;9:e78560. doi: 10.2196/78560 (PMC12829900; doi:10.2196/78560)
Supplement: Multimedia Appendix 1 — Survey instrument. [file nursing-v9-e78560-s001.docx]

Swan’s [28] survey had 46 content questions (e.g., asking about AI, and AI effects on nursing) plus a demographic section. Swan’s first question was not used. It was replaced by two questions to gain a sense of computer expertise [27] and acceptance of technology of the respondents. The remainder, i.e., 45 of Swan’s questions were used in this study, however some questions were amalgamated, and the questions were reordered. For example, the following 5 questions from Swan [28] became a single question in this current study.

- Were you aware of speech-text recognition from the media, TV, radio? (yes or no)
- Were you aware of speech-text recognition from social media? (yes or no)
- Were you aware of speech-text recognition form lectures at your college/university? (yes or no)
- Were you aware of speech-text recognition from your employment? (yes or no)
- Where you aware of speech-text recognition from family/friends? (yes or no)

This was adapted to:

How did you become aware of speech-text recognition? (check all that apply)

- from media, TV, radio
- from Social media
- from college or university lectures
- from your workplace
- from family and friends
- was not aware it was a form of artificial intelligence (added by the current authors)
- other, specify (added by the current authors)

Therefore, the revised survey tool used in this study had 37 content questions plus a demographic section and an optional “any additional comments”. The following table provides the survey divided into 8 sections, the survey questions grouped to align with the proposed research questions, the source of the survey question, whether survey question has been adapted, added or amalgamated and whether the survey question is similar to another previewed survey. The category ‘Similar to’ other previewed survey further supported that important topics were included.

|  | **Question** | **Response** | **Source** | **Similar**  **to** |
| --- | --- | --- | --- | --- |
| Part 1 –Insights on computer expertise and acceptance of technology | | | | |
| Q1 | Please chose the response that best describes your computer expertise | - Hardly ever use the computer and do not feel very competent. - Slightly below average computer user, infrequent using the computer using few applications. - Average computer user, using the internet, standard applications. - User of specialist application(s) but not an IT specialist - Considerable IT expertise short of full professional qualifications - Professionally qualified computer scientist or IT specialist | [27]  Question added |  |
| Q2 | Please indicate your level of agreement with the following statement: In general, I welcome new technology in my nursing practice. | - Strongly Agree - Agree - Neutral - Disagree - Strongly disagree | [current authors]  Question added |  |
| Part 2 - Main sources of knowledge for learning about current day-to-day AI | | | | |
| Q3 | How did you become aware of speech-text recognition? | - from media, TV, radio - from Social media - from college or university lectures - from your workplace - from family and friends - was not aware it was a form of artificial intelligence^a^ - other, specify^a^ | [28]  ^a^[current authors] Questions amalgamated, added to potential responses |  |
| Q4 | How did you become aware of spam-filters? | - from media, TV, radio - from Social media - from college or university lectures - from your workplace - from family and friends - was not aware it was a form of artificial intelligence^a^ - other, specify^a^ | [28]  ^a^[current authors] Questions amalgamated, added to potential responses |  |
| Q5 | How did you become aware of recommendation algorithms? | - from media, TV, radio - from Social media - from college or university lectures - from your workplace - from family and friends - was not aware it was a form of artificial intelligence^a^ - other, specify^a^ | [28]  ^a^[current authors] Questions amalgamated, added to potential responses |  |
| Part 3 – Description of their level of knowledge of AI technologies  Awareness of the emergence of AI or ML in nursing | | | | |
| Q6 | How would you describe your understanding of the technologies used in artificial intelligence? | - Excellent - Very Good - Good - Fair - None | [28] |  |
| Q7 | How would you describe your understanding of the technologies used in machine learning or deep learning? | - Excellent - Very Good - Good - Fair - None | [28]  [current authors], the term machine learning was added. | Similar to [31] |
|  | Are community RNs aware of the emergence of AI or ML in nursing? | | |  |
| Q8 | Indicate whether you have heard of artificial intelligence in healthcare or nursing. | - yes, healthcare - yes, nursing - no, healthcare - no, nursing | [28]  [current authors] Questions amalgamated. |  |
| Q9 | Indicate whether you have heard of machine learning or deep learning in healthcare or nursing. | - yes, healthcare - yes, nursing - no, healthcare - no, nursing | [28]  [current authors] Questions amalgamated, the term machine learning added. |  |
| Part 4 - Usefulness of AI applications Q10-Q21 | | | | |
| Q10 | Automated detection of an individual’s signs and symptoms would be useful. | 5_Strongly agree  4_Agree  3_Neutral  2_Disagree  1_Strongly disagree | [28] |  |
| Q11 | Automated assessment of social determinants of health would be useful. | As above – all the way down | [28] |  |
| Q12 | Automated outcomes prioritizing types of clients and targeting services to support care coordination would be useful. |  | [28] |  |
| Q13 | Automated outcomes to support the need for a different level of care (transition management) would be useful. |  | [28] |  |
| Q14 | Automated identification of problem areas or nursing diagnosis(ses) to support care planning would be useful. |  | [28] |  |
| Q15 | “Bots” that send client reminders would be useful. |  | [current authors]  Question added |  |
| Q16 | Automatic recommendations on the appropriate health assessment to perform such as geriatric assessment or screening for depression and anxiety, etc. would be useful. |  | [28] |  |
| Q17 | Automatic recommendations on the appropriate physical examination to complete such as vital signs, palpation, auscultation, etc. would be useful. |  | [28] |  |
| Q18 | Automated prediction of risk, such as falls, readmission to hospital, depression, mortality etc. with recommendations of appropriate nursing actions/interventions would be useful. |  | [28] |  |
| Q19 | Automated documentation of my nursing visit would be useful. |  | [28] |  |
| Q20 | Automated summarizing of specific relevant nursing topics from client’s narrative notes would be useful. |  | [28] |  |
| Q21 | Other applications? Please write in space below | Note: Not included in the Chi-square test because asking for other possible applications not identifying their agreement | [28] |  |
| Part 5 - Effects of AI on nursing practice Q22-25 | | | | |
|  | Used for Perceptions of effects of AI on nursing practice | | |  |
| Q22 | Artificial intelligence will revolutionize nursing by supporting health promotion and disease prevention, helping create personalized treatment plans, speeding up administrative tasks, automating routine tasks. | 5_Strongly agree  4_Agree  3_Neutral  2_Disagree  1_Strongly disagree | [28] |  |
| Q23 | Artificial intelligence will revolutionize healthcare by supervising population health management such as chronic disease surveillance, motivating individuals in healthy behaviours, focusing on prevention and less on intervention, decreasing costs, transforming care experience for individuals and providers. | As above – all the way down | [28] |  |
| Q24 | The human registered nurse will be replaced in the near future by an artificial intelligence nurse. |  | [28] | Similar to [30-32,34] |
| Q25 | Members of the interprofessional care team will be replaced in the near future by artificial intelligence team. |  | [28] |  |
| Part 6 - Perception of AI in general on their practice Q26-35 includes Accountability Q32-33 | | | | |
| Q26 | The developments in artificial intelligence, machine learning, deep learning, make me feel uncomfortable. | 5_Strongly agree  4_Agree  3_Neutral  2_Disagree  1_Strongly disagree | [28] | Similar to [26,31] |
| Q27 | The developments in artificial intelligence and deep learning, make nursing more exciting to me. | As above – all the way down | [28] | Similar to [26,30,31] |
| Q28 | The developments in artificial intelligence and deep learning, make healthcare more exciting to me. |  | [28] |  |
| Q29 | Artificial intelligence is part of nursing practice. |  | [28] | Similar to [31] |
| Q30 | Artificial intelligence should be part of nursing education. |  | [28] |  |
| Q31 | Artificial intelligence should be part of continuing professional development. |  | [28] |  |
| Q32 | I am concerned because it is not clear who becomes responsible if AI-based tools offer wrong recommendations to the nurse. |  | [36]  Question added | Similar to [30,32] |
| Q33 | I am concerned because it is not clear who is responsible if appropriate AI-recommendation options are mistakenly dismissed by the nurse. |  | [36]  Question added | As above other sources asked about accountability |
| Q34 | Nurses should be consulted in the integration of artificial intelligence that has implications on nursing practice. |  | [current authors]  Question added |  |
| Q35 | Nurses should be encouraged to identify relevant nursing questions that would benefit from artificial intelligence to support their decision-making. |  | [current authors]  Question added |  |
| Part 7 – Identification of competencies  How to involve nurses | | | | |
|  | Competencies | |  |  |
| Q36 | Identify the artificial intelligence competencies that each group of nurses (i.e., nursing students, registered nurses, researcher/faculty) should possess? Check all that apply. | - Knowledge of common types of artificial intelligence - Knowledge of common uses and outcomes of artificial intelligence - Knowledge of benefits/limitations when considering the different types of artificial intelligence^a^ - Knowledge how machine learning can improve processes and outcomes, for example, augmenting human decision-making - Knowledge and skills in integrating human and artificial intelligence reasoning (logic). - Skills to support information management platforms, for example, big data - Statistical knowledge and skills related to clinical analytics, data management, and algorithm awareness. - Leadership and emotional intelligence to embrace a transformed healthcare delivery system where artificial intelligence is making every day operational decisions. - Problem-solving ability and judgement-based solutioning to construct questions in a way that artificial intelligence technologies will be able to process and create responses that correctly guide decisions. - Communication, collaboration, and cross-functional knowledge - Other, Specific | [28]  ^a^[current authors] competency added  Amalgamated into a multiple response question. |  |
|  | Used to gain insights on how to involve nurses. | |  |  |
| Q37 | How should nurses be involved in artificial intelligence that influences their practice? | Free text | [current authors] |  |
| Part 8 - Demographics | | | | |
| Q38 | Demographics | - What year were you born? - What is your gender? - Please specify the Province/Territory where you are currently employed? - Please specify your highest level of education. - Please specify your current position (multiple response Q) - Please specify your community practice setting, - What sector employs you? - How many years have you worked as a registered nurse? - How many years have you worked as a registered nurse providing care in a community setting? | [28]  [current authors]  Adapted to reflect Canadian terms |  |
| Q39 | Optional: Any addition comments you would like to make about this study? | - Free text |  |  |

^a^Current authors

References

1. Schepman A, Rodway P. Initial validation of the general attitudes towards Artificial Intelligence Scale. Computers in human behavior reports. 2020 Jan 1;1:100014.
2. Schepman A, Rodway P. The General Attitudes towards Artificial Intelligence Scale (GAAIS): Confirmatory validation and associations with personality, corporate distrust, and general trust. International Journal of Human–Computer Interaction. 2023 Aug 9;39(13):2724-41.
3. Swan BA. Assessing the Knowledge and Attitudes of Registered Nurses about Artificial Intelligence in Nursing and Health Care. Nursing Economic$. 2021 May 1;39(3).
4. Botwe BO, Antwi WK, Arkoh S, Akudjedu TN. Radiographers’ perspectives on the emerging integration of artificial intelligence into diagnostic imaging: The Ghana study. Journal of medical radiation sciences. 2021 Sep;68(3):260-8.
5. Coakley S, Young R, Moore N, England A, O'Mahony A, O'Connor OJ, Maher M, McEntee MF. Radiographers’ knowledge, attitudes and expectations of artificial intelligence in medical imaging. Radiography. 2022 Nov 1;28(4):943-8.
6. Oh S, Kim JH, Choi SW, Lee HJ, Hong J, Kwon SH. Physician confidence in artificial intelligence: an online mobile survey. Journal of medical Internet research. 2019 Mar 25;21(3):e12422.
7. Abdullah R, Fakieh B. Health care employees’ perceptions of the use of artificial intelligence applications: survey study. Journal of medical Internet research. 2020 May 14;22(5):e17620.
8. Esmaeilzadeh P. Use of AI-based tools for healthcare purposes: a survey study from consumers’ perspectives. BMC medical informatics and decision making. 2020 Dec;20:1-9.
